# Evaluation of Flexible and Integrative Psychiatric Treatment Models in Germany—A Mixed-Method Patient and Staff-Oriented Exploratory Study

**DOI:** 10.3389/fpsyt.2018.00785

**Published:** 2019-01-22

**Authors:** Sebastian von Peter, Yuriy Ignatyev, Jakob Johne, Sonja Indefrey, Onur Alp Kankaya, Burkhard Rehr, Manfred Zeipert, Andreas Bechdolf, Thomas Birkner, Arno Deister, Annette Duve, Sandeep Rout, Harald Scherk, Anna Schulz-Dubois, Bettina Wilms, Dyrk Zedlick, Peter Grollich, Bernard Braun, Jürgen Timm, Martin Heinze

**Affiliations:** ^1^Department of Psychiatry and Psychotherapy, Brandenburg Medical School Theodor Fontane, Immanuel Klinik Rüdersdorf, Rüdersdorf, Germany; ^2^Department of Psychiatry and Psychotherapy, Charité University Medicine Berlin, Berlin, Germany; ^3^Department of Psychiatry and Psychotherapy, Vivantes Krankenhaus am Urban, Charité University Medicine Berlin, University of Cologne, Berlin, Germany; ^4^Department for Psychiatry, Psychotherapy and Psychosomatic Medicine, Westklinikum Heide, Heide, Germany; ^5^Psychosoziales Zentrum Itzehoe, Itzehoe, Germany; ^6^Department of Child and Adolescent Psychiatry, Vitos Klinikum Riedstadt, Riedstadt, Germany; ^7^Department of Psychiatry and Psychotherapy, Vivantes Krankenhaus Neukölln, Charité University Medicine Berlin, Berlin, Germany; ^8^Department of Psychiatry and Psychotherapy, Vitos Klinikum Riedstadt, Riedstadt, Germany; ^9^Department of Psychiatry and Psychotherapy, Imland Krankenhaus Rendsburg, Rendsburg, Germany; ^10^Department of Psychiatry and Psychotherapy, Basedow Klinikum Saalekreis, Querfurt, Germany; ^11^Department of Psychiatry and Psychotherapy, Rudolf Virchow Krankenhaus Glauchau, Glauchau, Germany; ^12^SOCIUM Research Center, University of Bremen, Bremen, Germany; ^13^Biometry Section, Competence Center for Clinical Trials, University of Bremen, Bremen, Germany

**Keywords:** implementation, cross sectoral mental health care, user evaluation, staff evaluation, mixed method, regional budget, block contract, capitation

## Abstract

Contrary to the practice in some countries, access to flexible and integrated forms of psychiatric care (FIT models) is limited in Germany. Several legislations have been introduced to improve this situation, notably the recent §64b (flexible and integrative treatment model; FIT64b) of the German Social Code, which allows for a capitation-based accounting of fees for services. The aim of this study was to explore the effects of FIT64b implementation on various stakeholders (patients, informal caregivers and staff) in 12 psychiatric hospital departments across Germany. Structural as well as quantitative and qualitative data are included, with integration of different methodological approaches. In all departments, the implementation of the new accounting system resulted into a relatively stable set of structural and processual changes where rigid forms of mainly inpatient care shifted to more flexible and integrated types of outpatient and outreach treatments. These changes were more likely to be perceived by patients and staff, and likewise received better evaluations, in those departments showing higher level or longer duration of implementation. Patients' evaluations, furthermore, were largely influenced by the advent of continuous forms of care, better accessibility, and by their degree of autonomy in steering of their services.

## Introduction

Internationally, there is near consensus that community-based integrated and comprehensive psychiatric services performed by interdisciplinary teams constitutes the gold-standard for the care of patients suffering from mental illness. In addition to community mental health treatment (CMHT), various forms of more integrated approaches have been developed for special purposes. These include Crisis Resolution Teams (CRT), Assertive Community Treatment (ACT), and Intensive Case-management in conjunction with Home-Treatment programs (ICM and HT) (1–6).

Yet, despite good evidence for their effectiveness (3, 5), these programs are not part of standard psychiatric care in Germany. The treatment paradigm in Germany is characterized by a relatively large proportion of in-patients (7), and a rather deficient integration of in-patient-services with out-patient services, office-based psychiatry, and psychotherapy, and with a broad spectrum of other psychosocial institutions (7, 8). Further, current reimbursement practices do not incentivize the integration of these sectors and treatment settings (7): Around 140 health insurance companies -both statutory and private- and various, mostly public funding agencies cover a wide spectrum of expenditures, leading to a situation lacking in integration. As such, the German system is sometimes described as being highly fragmented (7) and particularly lacking in access to outreach services (9).

Different legislations aiming at improving this state of affairs and changing the incentives in the current mental health care system have been introduced in the German Social Security Code over the last two decades. The goal of these legislations was mainly to facilitate the bridging between various sectors, and particularly between in- and out-patient forms of care. In addition, legislation aimed at a more rational use of resources, based upon the assumption that the fragmented nature of the German mental health care system also leads to wastage. Many of these legislations allowed for the use of either capitation-based funding approaches or block contracts that both aim at incentivizing cooperation across various sectors and institutions (10). This resulted into various forms of flexible and integrative treatment (FIT) models, many of which being hospital-based, such as the well-known regional budget (11–14) or specific home treatment programs (15–17). In addition, recent years have seen the establishment of integrated care programs within both hospital and community mental health institutions (18–20). To summarize, since they make use of different forms of social regulations, FIT models are diverse and difficult to compare, which impedes their evaluation against standard systems of care. The most recent FIT innovation stems from §64b in the revised German Social Code V (= FIT64b programs) (21). This legislation aims to encourage new models of integrated and flexible care for the mentally ill by enabling cross-sectoral service delivery and complex outpatient forms of psychiatric treatment, in both the clinic and home-environments. A fixed total budget is allocated to the service providers that is meant to cover all forms care, i.e., an application of the fee capitation principle or block contracts (10, 13, 22). This budget is paid once a year and must cover all expenses, while leaving sufficient latitude to the service provider for adapting treatments to the needs of a region or individual patients. The funding is not confined to specific activities, such that the service provider is free to allocate resources and to offer various forms of treatment

FIT64b-projects in Germany are currently offered only within the hospital sector, involving a transformation from previously daily and bed-related hospital rates to block contract and capitation reimbursement systems. According to the law, they have an experimental character, being restricted to a maximum duration of 8 years. Based on outcome research after this trial period, the German government will decide if this approach should properly become a permanent part of the standard medical system. We recognize that many other models have been implemented and evaluated, but focus our present investigation on models resulting from §64b SGB-V (FIT64b). We feel that a detailed examination of FIT64b models should generalize to discussions on the benefits of other FIT models in general, and, even more broadly, on the effects of block contracts and capitation-related systems of reimbursement.

A total of 19 FIT64b projects are presently underway across various hospital departments in Germany, which differ considerably in terms of duration of services, contextual settings, treatment structures and processes, all depending on historical contingencies and local circumstances (23). At the same time, these projects all seek to offer continuous, flexible, and integrated models of care rather than the traditionally rather brief and rigid sets of mainly inpatient treatment. Moreover, existing FI64b projects entail complex interventions encompassing several interacting components, thus requiring a mixed method and multi-phase assessment model, including a substantial element of process evaluation, for assessing their multifactorial effects (24, 25).

A careful evaluation of FIT64b projects is a matter of scientific interest in addition to its legal implications. Evidence-based evaluation of performance is crucial for their assessment. As mandated by law, there is an on-going evaluation study that is financed by the health insurance companies themselves (EvaMod) (26). Yet, this evaluation concentrates on only routine hospital data and economic analyses. In contrast, it does not involve any stakeholder-centered outcome evaluation. This raised questions within the scientific community about the fitness of this EvaMod study for the comprehensive evaluation of FIT-64b models. Consequently, various hospital departments commissioned and financed the present study (“EvaMod64b”), which is meant to be a supplement to EvaMod, also involving the experiences and evaluations of the various stakeholder concerned.

The aim of this study was to explore the multi-variant effects of 12 FIT64b hospital psychiatry departments across Germany on various stakeholders (patients, informal caregivers and staff). To enable a multi-faceted analysis, we aimed to consider the several stakeholders' experiences and evaluations and the phase of implementation of each FIT64b project. To meet this objective, we included structural, and quantitative and qualitative data from all three stakeholder groups, while integrating the different methodological approaches in a single model. Due to large differences in FIT64b practices between the 12 sites, we had to implement new strategies for integrating data sources. A description of our methodological challenges can be found elsewhere (27–29); we now report the main results and conclusions of the multi-center and mixed method evaluation study “EvaMod64b.”

## Materials and Methods

Ten hospitals with FIT64b models pooled their resources to fund the evaluative study “EvaMod64b.” The study was approved by the Ethics Committee Brandenburg [2016, No. S 7 (a)], thus adhering to the ethical standards laid down in the 1964 Declaration of Helsinki and its later amendments. All eligible patients were given a comprehensive description of the project, were informed that their participation or refusal would not affect their care, and provided written consent, with guaranteed anonymity. Prior to the main assessment we undertook preliminary exploratory studies to optimize study materials (28, 29).

### Setting and Sampling

In 2015, leaders of the 15 then-established FIT64b projects where invited to participate in the multicenter study, of which 13 departments agreed (ten adult psychiatry and three child and adolescent psychiatry units in Itzehoe, Heide, Rendsburg, Lüneburg, Nordhausen (both adult and child/ adolescent psychiatry), Glauchau, Riedstadt (both adult and child and adolescent psychiatry), Rüdersdorf, and Berlin (with adult psychiatry in Kreuzberg/Friedrichshain and Neukölln, and child/adolescent psychiatry in Friedrichshain). The start date of FIT models ranged from January 2013 to January 2016. Six departments had a history of FIT within frameworks of other social regulations, either according to the regulations for regional budget or integrated care programs.

Of the 13 departments, for organizational reasons one department withdrew from the study. The remaining 12 departments contributed sets of structural data and data for process analyses from all three stakeholder groups. Further, we restricted this report to the ten adult departments, with results of the two child/adolescent departments to be reported in another publication. In addition, the response rate of informal caregivers was too low to support productive qualitative and quantitative findings, such that related data were omitted from further analyses. Because of the considerable heterogeneity of specialized therapists' professional backgrounds and fields of activities, we confined our analysis to data provided by physicians/psychologists and nurses. Finally, we excluded the staff and patient-related data from three departments, as these projects had sparse reporting of the FIT64b data. The ten included projects provided data representative of their specific mix of treatment approaches, some of which received or offered traditional forms of care, and others utilizing only FIT64b-specific treatments.

**Figure 1:**
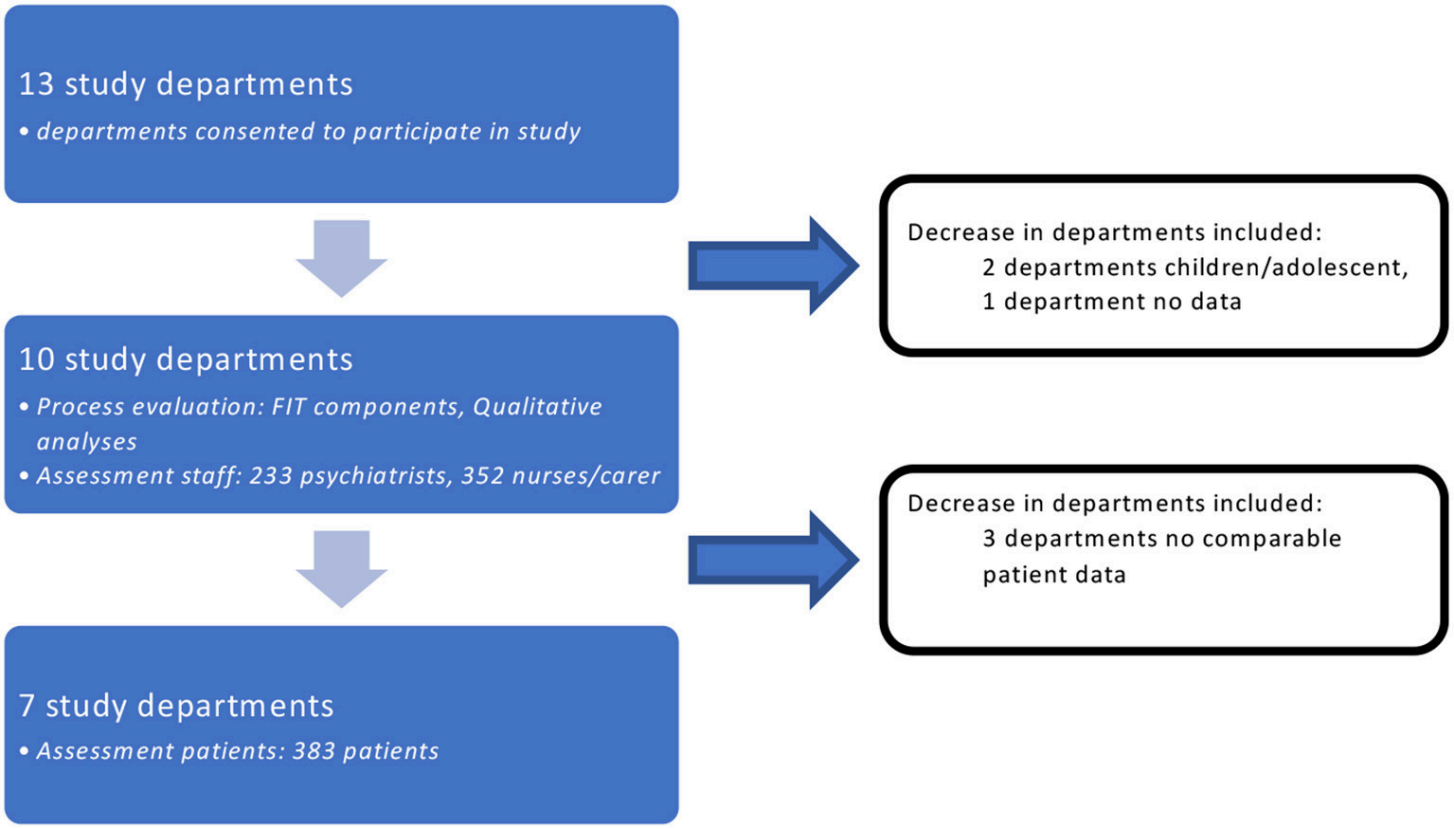
Departments involved (Study flow).

In summary (see Figure 1), we present herein our findings for adult psychiatric departments only. Process and structure-related analyses, and likewise the assessment of staff' evaluations and experiences refer to the ten included FIT64b departments, whereas the analyses of patients' experiences and evaluations refer to only seven FIT64b departments.

### Grading of FIT64b Implementation of Mental Health Departments

The participating departments were compared using structural and statistical data such as duration and previous history of FIT64b projects, their organizational structure, size of catchment area, departmental size and annual caseloads, average length of in-patient stay, statistical breakdown in involved insurance companies, and the proportions of in- and outpatient care.

To assess FIT64b differences between mental health departments, we identified a set of quantified program components and the total score of these components, reflecting the degree of FIT-64b implementation in each department (27, 29). This was accomplished using a complex, multi-step and iterative research process following the Grounded Theory Methodology (30). The model included 11 components (C), as follows: (C1) shifting from in- to outpatient settings, (C2) flexible care management across settings, (C3) continuity of care, (C4) multi-professional cooperation, (C5) therapeutic group sessions across settings, (C6) outreach care, (C7) involvement of informal caregivers, (C8) accessibility of services, (C9) patient autonomy in steering of services, (C10) cooperation across sectors, and (C11) growth of professional expertise. Components were operationalized and quantified (28, 29), such that comparing the various departments became possible. We identify below the 11 FIT64b process components numerically and the total score as FIT64b-total.

### Qualitative Process and Outcome Evaluation

Thirty one focus groups (31) and 15 expert interviews (32), including a total of 63 patients and 138 staff, were carried out across the ten included FIT64b projects. Sampling was plotted using various criteria that were relevant for forming or influencing the experiences with FIT64b treatment models (33). For inclusion criteria, see section Measuring Patients' Experience and Evaluation.

We developed 14 research guidelines in 11 thematic fields to carry out qualitative evaluation of the FIT64b components. The guidelines involved questions about the perceived benefits and disadvantages of specific FIT64b structures and processes. Our aim here was to collect data on how the changes of treatment routines upon implementation of an FIT64b model were experienced by staff and patients. Data were analyzed by content analyses (34), beginning with a process of open decoding and proceeding to include the above-mentioned FIT64b components as main deductive categories. Coding was performed by the research team and by two coders per transcript. Results were ordered according to contents of the components that were also used in the quantitative part of the study, described below.

Qualitative data were used both for carrying out a substantial process evaluation (35) and in assessing perceived effects for patients and staff of implementing the FIT64b projects (qualitative outcome evaluation). Analysis of clinical data and grading of adherence to FIT64b added to the process evaluation, thus helping to relate qualitative and quantitative outcomes and to assess effectiveness of the FIT64b models.

### Measuring Staff' Experience and Evaluation

Upon obtaining approval from the respective works councils, a standardized written survey of physicians/psychologists, nurses, and specialized therapists was made between October 2016 and February 2017. The core element of the analysis was a specific 27-item scale that was based on the above-mentioned 11 FIT64b components. The following key question was posed for the 27-item scale to judge the grade of implementation as perceived by staff members: “How do you rate the impact of such structures und procedures for the treatment and care for patients with mental illness in your hospital such as are already partially realized and enabled by FIT64b on the outcomes of your occupational routine in the last months?” In the first part, permitted responses about staff members experience were “non-existent,” “existing, but not yet assessable,” or “existing and assessable.” In the second part, staff member evaluations for each item were “very positive,” “rather positive,” “partly,” “rather negative,” or “very negative.” Moreover, there were four additional questions concerning possible impacts on working conditions (e.g., increase in overtime) and 13 statements about FIT64b, such as “FIT64b leads to less bureaucracy and increased professional autonomy.”

In addition to these FIT64b-specific aspects, participants were asked to rate their present working situation. We adopted 23 questions for physicians/psychologists and 27 for nurses with slight modifications from the German “Questionnaire on Working Situation for Doctors” (FAÄ) and the corresponding questionnaire for nurses” (FAP), based upon repeatedly tested and validated items from other research projects in comparable settings (36). We also added questions about the extent of negligence of health-related well-being and about implicit rationing of treatments/services. The question on possible implicit rationing had been validated in the international research project “Registered Nurses Forecast” (RN4CAST) (37). Information about structural aspects of the workplaces (e.g., type of department, setting, and number of patients), sociodemographic factors (e.g., age) as well as professional aspects of respondents (e.g., vocational training, occupational period in job and hospital) were requested.

### Measuring Patients' Experience and Evaluation

The patient sampling was conducted using equally sized patient cohorts from different care sectors (wards, day hospitals, outpatient clinics, or outreach care). The recruitment process within each care sector was based on a randomized design. The inclusion criteria were: age ≥ 18 years, capacity to provide informed consent, ability to read and understand German. Patients were excluded if their comprehension was limited by acute mental disorders or severe mental disability, as judged by their psychiatrist, or if their admission to the clinic was involuntary.

To assess patient experiences and evaluation, we used the in-house survey, Scale for Evaluation of Psychiatric Integrative and Continuous Care (SEPICC) (28). This scale consists of two sections; part one assesses the patients' experiences with several FIT64b components and part two entails their evaluations. The possible scores for each answer range from 0 (not at all true) to 4 (entirely true), where 2 indicates a neutral opinion. Furthermore, the SEPICC concept allows one to estimate the consistency of a patient's answers by posing contradictory questions concerning two aspects of FIT64b (questions 3 and 8 vs. 5 and 10). Based on a grading of concordance for these pairs of answers, the consistency of the patient responses was indicated by a score ranging from 0 to 1.

The summarized patient's experience score was represented as EXPtotal, whereas the summarized patient's evaluations of these experiences were designated as EVAtotal. To assess current psychopathology, we used a short version of the SCL-90-R (38). These questionnaires were filled out by the participants without assistance. Additionally, some socio-demographic and clinical characteristics (gender, age, education status, employment status, family status, and duration of the current mental disorder) were recorded (see Table 2 for items documented).

### Statistical Analysis

We calculated descriptive statistics to assess clinical and demographical data, quantitative experiences, and evaluations by patients and staff. For exploring trends of these parameters vs. emerging FIT64b components, we applied bivariate analysis. These analyses cover trends of experience (EXPtotal) and evaluation (EVAtotal) of patients and therapeutic staff regarding FIT64btotal or its 11 components (C1-C11). Additionally, we analyzed the different project time durations as well as the association between experience and evaluation scores. Trends were tested by a non-parametric Jonckheere test. Group differences were examined with Kruskal-Wallis and continuous parameters with the Mann-Whitney test. Categorical data were tested using the χ^2^ test or (in case of small cell counts) Fisher's exact test. All exploratory tests used alpha = 5%, and any test result with alpha < *p* < 2^*^alpha was deemed significant.

Based on the measures and scores defined in sections Grading of FIT64b Implementation of Mental Health Departments and Measuring Patients' Experience and Evaluation, the patient-oriented goal of the study might be expressed in detail by the following two primary working hypotheses. First, FIT64b oriented process development in departments will result in higher FIT64b-specific experience scores of involved patients. Thus, we predicted a trend of increasing experiences, measured by EXPtotal, with increasing implementation of FIT64b components, measured by FIT64btotal. Second, patients will give higher evaluation of FIT64b settings if their department is more compliant with FIT64b components. The corresponding null-hypotheses can be stated as “EXPtotal independent of FIT64btotal score” and “EVAtotal independent of FIT64btotal score.” Both hypotheses can be expanded by considering the individual component gradings instead of FIT64btotal. A secondary patient-oriented objective was the analysis of patient evaluations in relation to patient experiences with the various aspects of FIT64b. The corresponding null-hypothesis might be formulated by “EVAtotal is independent of EXPtotal scores.” Both primary working hypotheses were tested deductively and the secondary hypotheses in an exploratory manner. The primary alpha was adjusted to 2.5% since two deductive primary tests were performed.

All other analyses were declared as secondary and their statistical tests interpreted as explorative only, such that alpha = 5% was used for these analyses. Statistical results were computed by SAS 9.4 and Systat 13, and calculation of power for case numbers by nQuery+nTerim 2.0 and SPSS 15.0.1.

## Results

### Process Evaluation

#### Structural Data of Departments

The included clinics were either at public (seven departments) or non-profit (three departments) hospitals, regional population with regional catchments ranging from 85,000 up to 425,000 people. The hospitals furnished of 0.38–0.65 psychiatric beds and 0.13–0.37 day-clinic treatment places per 1,000 people. Of all patients treated according to §64b, groups of 27–72% patients received inpatient care and 33–72% received outpatient care during 2016. Six of ten examined departments had a previous history of FIT according to above-mentioned regulations other than §64b. Six projects had a duration of the FIT64b process >2 years and four projects had a briefer duration of ≤ 2 years. Four departments signed contracts with only one or two health insurance companies, and the remaining six were under contract with various companies.

#### Grading of FIT64b Implementation

All ten departments were assessed using the developed FIT64b components, yielding a range of implementations with FIT64btotal extending from 0.63 to 1.73 (mean 1.1 ± 0.35; Table 1). The trends of FIT64b components in projects with briefer (≤2 years) and longer (>2 years) duration showed that FIT64b components were more completely implemented in projects after 2 years of implementation (Table 1). Differences in the contrast were significant for C2 (*p* = 0.033) only, but FIT64btotal (*p* = 0.55) and C8 (*p* = 0.065) presented differences at 5% < *p* < 10%.

**Table 1:** Operationalization of FIT components.

**No**.	**Component**	**Operationalization**	**Assessment**
I	Shifting in- to outpatient setting *Shift of treatment from I^a^ toward D^b^ and/or O^c^*	• Number of outpatient SoF^d^/total number SoF^d^ during EP^e^	
II	Flexible care management across settings *Unproblematic shift of SoF^d^ (prompt, little bureaucracy*	• Number of CoT^f^ using all three SoF^d^ during EP^e^/total number CoT^f^ •Treatment D^b^, I^a^, and/or O^c^ in the same unit (ward, level etc.) •Systematic steering of treatment beyond all SoFs^d^ •Application of SoF^d^ spanning roster and therapy plans	Rating scale (0–2)
		• Number SoF^d^-spanning sessions (meetings etc.)	Rating scale (1–3)
III	Continuity of treatment team *Implementation of team- and individual-related continuity*	• Percentage of staff working in more than one SoF^d^ (on a regular basis) •Coordinated admission (coordinating staff member) • Coordination of treatment by e.g., case manager, SoF^d^-spanning care • Home treatment by I^a^- and D^b^- teams • Outsourced PIA (outpatient department) team (not working in I^a^ or D^b^)	Rating scale (0–2)
IV	Multiprofessional Cooperation *Intense multiprofessional cooperation*	• Absolute number of mandatory sessions across all occupational groups	Absolute number
		• Measure/action to optimize cooperation across all occupational group	Rating scale (0–1)
		• Training sessions multiprofessional cooperation	
		• Number occupational groups working in home treatment (on a regular basis)	Rating scale (0–2)
V	Therapeutic group sessions across all settings *Therapeutic groups with members from all SoF^d^*	• Number of group sessions open for all SoFs^d^	Rating scale (0–2)
VI	Outreach home care *Multiprofessional treatment at home ≥ 1 week*	• Number CoT^f^ with home-treatment/ all I^a^-cases during EP^e^	
		• Cars for home-visits	Rating scale (0–2)
VII	Involvement of informal caregivers *Informal caregivers as therapeutic tool*	• “Network” or other forms of systemic dialog with informal caregivers and/or “carer-conference” and/or “caregiver groups”	Rating scale (0–1)
		• Number of groups open for informal caregivers	Rating scale (0–1)
		• Percentage of systemic training for staff/employees (e.g., open dialogue)	Percentage
VIII	Accessibility of services *Geographical accessibility and accessibility of teams*	• Accessibility of services within 1-h drive • 24-h-accessibility of multiprofessional mental health team (not doctor on call or the like) • Shuttle service for services users	Rating scale (0–2)
		• Waiting list	Reverse rating scale (1–0)
IX	Sovereign steering of services *Freedom of therapeutic decisions*	• Number of exeats ≥ 2 nights in a row/all exeats during EP • Number of exeats per service user/calendar week during EP • Daypatient treatment as well during the night • Rules according to contract in all matters concerning setting of treatment and length of treatment	Rating scale (0–2)
X	Cooperation across Sectors *Cooperation with ambulant care systems*	• Mutual scheduling and realizing of treatment with ambulant care systems (SGB V) • Mutual scheduling and realizing of treatment with social welfare system (SGB XII)	Rating scale (0–2)
		• “Community psychiatric network”	Rating scale (0–1)
XI	Expansion of professional expertise *Professionalization of staff*	• Multiprofessional training of staff concerning FIT models • Measures to multiply knowledge about FIT models • FIT models as part of appraisal interviews	Rating scale (0–1)
		• Percentage of nurses/caregivers moderating group sessions	Percentage

a I, inpatient;

b D, day-patient;

c O, outpatient;

d SoF, setting of treatment (outpatient, day-patient, inpatient);

e EP, evaluation period;

f *CoT, case of treatment*.

**Table 2:** FIT64b component values for new, more established, and all FIT64b departments.

	***N***		**FIT64 total**	**C2**^a^****	**C3**^b^****	**C4**^c^****	**C5**^d^****	**C7**^e^****	**C8**^f^****	**C9**^g^****	**C10**^h^****	**C11**^i^****
FIT64btotal (**10 departments**)	**10**	**Mean**	**1.11**	2.14	**0.65**	2.40	**2.20**	0.51	**0.66**	**0.72**	0.63	0.90
		**std**	**0.35**	1.02	**0.38**	0.98	**1.01**	0.30	**0.24**	**0.34**	0.43	0.28
Test result	**10**	***p***	**0.055**	0.033	0.240	0.286	**0.492**	0.818	**0.065**	**0.724**	0.103	0.068
“Old” FIT64b projects (duration >2 years)	**6**	**Mean**	**1.28**	2.63	**0.78**	2.69	**2.50**	0.51	**0.77**	**0.77**	0.77	1.03
		**std**	**0.31**	0.98	**0.34**	1.03	**1.18**	0.32	**0.17**	**0.25**	0.46	0.31
“New” FIT64b projects (duration ≤ 2 years)	**4**	**Mean**	**0.86**	**1.41**	**0.45**	1.96	**1.75**	0.50	**0.49**	**0.64**	0.41	0.71
		**std**	**0.23**	**0.57**	**0.40**	0.81	**0.50**	0.31	**0.25**	**0.48**	0.31	0.12

*The table shows the total grade of implementation of each component across departments. (10 departments; std, standard deviation; test, Kruskal Wallis test results new vs. old; significant results in bold)*.

a *flexible care management across settings*,

b *continuity of care*,

c *multi-professional cooperation*,

d *therapeutic group sessions across settings*,

e *involvement of informal caregivers*,

f *accessibility of services*,

g *patient autonomy in steering of services*,

h *cooperation across sectors*,

i *growth of professional expertise*.

#### Time Lines and Obstacles of Implementation

Qualitative process evaluation revealed two phases of implementation of FIT64b models. We designate the first as the “departure phase,” which usually entails the first 2 years and manifests in a drastic reduction of number of psychiatric hospital beds. In this early phase, routinized processes and structural changes are put to the test, and new concepts are developed that require some adaptation of care workers' attitudes and practices. Changes during this phase are well recognizable for both staff and patients, whereas the changes commencing in year three of implementation comprise a second “plateau phase,” where the proportion of ambulatory patients increases, new treatment concepts are processed, and staff continuously develop their expertise.

We found that implementation of FIT64b models faced several obstacles, especially when therapeutic concepts had either to be adapted or newly developed. For instance, new concepts for night clinic treatments and emergence of flexible teams charged with both out- and inpatient treatment appeared along with FIT64b implementation (material coded within C2 and C3). Further, previous subdivisions of wards proved to be no longer functional. In some departments, new buildings had to be constructed to meet the demands for flexible and continuous forms of treatment (C2 and C3). Manpower and work shifts had to be reorganized, requiring new IT-solutions supportive of continuous forms of care (C3). Finally, staff of various disciplines had to acquire new expertise (C11), i.e., how to more effectively treat patients as outpatients and assume new responsibilities. Care providers had to change customary attitudes that had been stabilized for years to act more flexibly in treating patients in more cooperative and trustful ways. Accordingly, new forms of financial compensation for changed work profiles had to implemented.

### Experience and Evaluation by Therapeutic Staff

#### Quantitative Analyses

Quantitative analysis of staff experience and evaluation refers to ten FIT64b projects. The entire data set consisted of 200 evaluable questionnaires from physicians/psychologists (response rate 31–82%, mean 60.2%, SD 14.8) and 308 from nurses (response rate = 20–87%, mean 42.0%, SD 21.4). Selected sociodemographic data showed a mean age of 39.7 years for physicians/psychologists and 44.3 years for nurses, an over-representation of female staff (68.3% for physicians/psychologists and 71.6% for nurses) and a mean work experience in psychiatry of 9.7 years (SD 9.3) for physicians/psychologists and 16.0 years (*SD* = 9.8) for nurses. Most staff were employed full time (61.8% for physicians/psychologists and 66.5% for nurses). Among the physicians, 29.4% were in assistant positions, and 17.9% were senior physicians. Most of the nurses (86%) had more than 3 years of professional education, and 50.5% of staff was working in general psychiatry, of whom 53% in inpatient and 73% also in outpatient settings.

Bivariate analyzes of the possible associations between the degree of implementation, as measured by the above-mentioned key questions and via selected structural and personnel characteristics of staff, showed no statistically significant associations. However, positive evaluations of physicians/psychologists were more frequent (59.6%) in projects with longer duration (>2 years), compared to projects with duration less than 2 years) (38.5%; χ^2^ = 8.869; *f* = 1; *p* = 0.002). There was a similar significant trend (χ^2^ = 10.090; *df* = 1; *p* = 0.001) for nurses, who had 17.7% positive evaluations for brief duration and 34.2% for longer duration. The grade of implementation as measured by the surveys key question differed between physicians/psychologists and nurses: Whereas 49.5% of physicians/psychologists rated the 27 FIT64b-items as “very” or “rather positive,” only 27.6% of nurses did so. Among responding physicians/psychologists 16.6% and among nurses 27.5% didn't rate any of the 27 items as positive.

#### Qualitative Analyses

High saturation of qualitative data was yielded for C1, C2, C3, C6, C9. Overall, staff perceived FIT-64b models to considerably “improve therapeutic relationships” (reference to coded material: FG_E1:10), allowing for long-term interactions (material coded along with C2) across settings (C3) and even treatment at home (C6), all of which was perceived as leading to a “more complete impression of the patient's situation” (FG_V2:15, FG_Z8.1:13, I_Z2.2:26, FG_Z3:16). Staff declared that interventions could be adapted better to the patient's needs (C2 and C9). FIT64b models were perceived to “offer more therapeutic options” (FG_E1:3), while at the same time “rendering daily routines more complex” (C3) (FG_V6:18, I_M2:14) and requiring an extra commitment of time-intensive multi-professional cooperation (C4).

In some clinics, the budgetary system of FIT64b models was perceived to reduce administrative demands (C9), whereas in others, particularly clinics that has signed contracts with only one health insurance company, the organizational requirements were deemed to have increased substantially. Overall, and in accordance with quantitative results, staff described an “increased work load” (I_Z3:13), occurring mainly during the implementation phase, but also persisting due to the expertise and responsibilities (C11) required for the new forms of outpatients (C1) and outreach treatments (C6). Yet, this evaluation was ambivalent, as these new responsibilities were also perceived to be “empowering and motivating” (FG_Z5:16; FG_M5:19), especially for those professional groups with what has traditionally been a more subordinate role.

### Experience and Evaluation by Patients

#### Quantitative Analyses

This part of our study entails findings from seven departments and 383 patient questionnaires. The majority (66.8%) of the patients were female and the mean age was 45.8 ± 14.7 years. The patients suffered from mental illness of mean duration 10.2 ± 10.9 years. On the SCL9K scale, the mean severity score was graded as 1.69 ± 0.89. 38.4% of the patients were single and 44.2% lived with a partner. Of the patients, 35.2% had a secondary, and 44.2% a post-secondary education: 23.3% of the patients were unemployed, 32.4% employed, and 32.4% retired.

Furthermore, patients were asked about their present therapeutic settings: A total of 138 patients (36.0%) received outpatient treatment, 113 (29.5%) were in a day clinic, 122 (31.9%) received treatment on a ward, and 20 (5.2%) received outreach care.

##### Experiences of patients

The analysis of the patients' FIT64b experiences using the SEPICC questionnaire yielded 236 (63.8%) who reported experiencing a flexible shift of settings (C3), whereas 227 (59.3%) had received treatments in different settings. 105 (46.3%) of these patients had been treated by the same team (C3), 222 (58.0%) had experienced mixed therapeutic groups (C5), and 171 (45.8%) had experienced a broadening expertise of staff (C11). 46 (12.0%) of the patients had received outreach care (C6), of whom 36 (78.3%) had experienced these forms of care for more than 1 week. The total of experiences as summarized in a combined score EXPtotal ranged from 0 to 9, with a mean total score of 3.75 ± 1.88.

**Figure 2:**
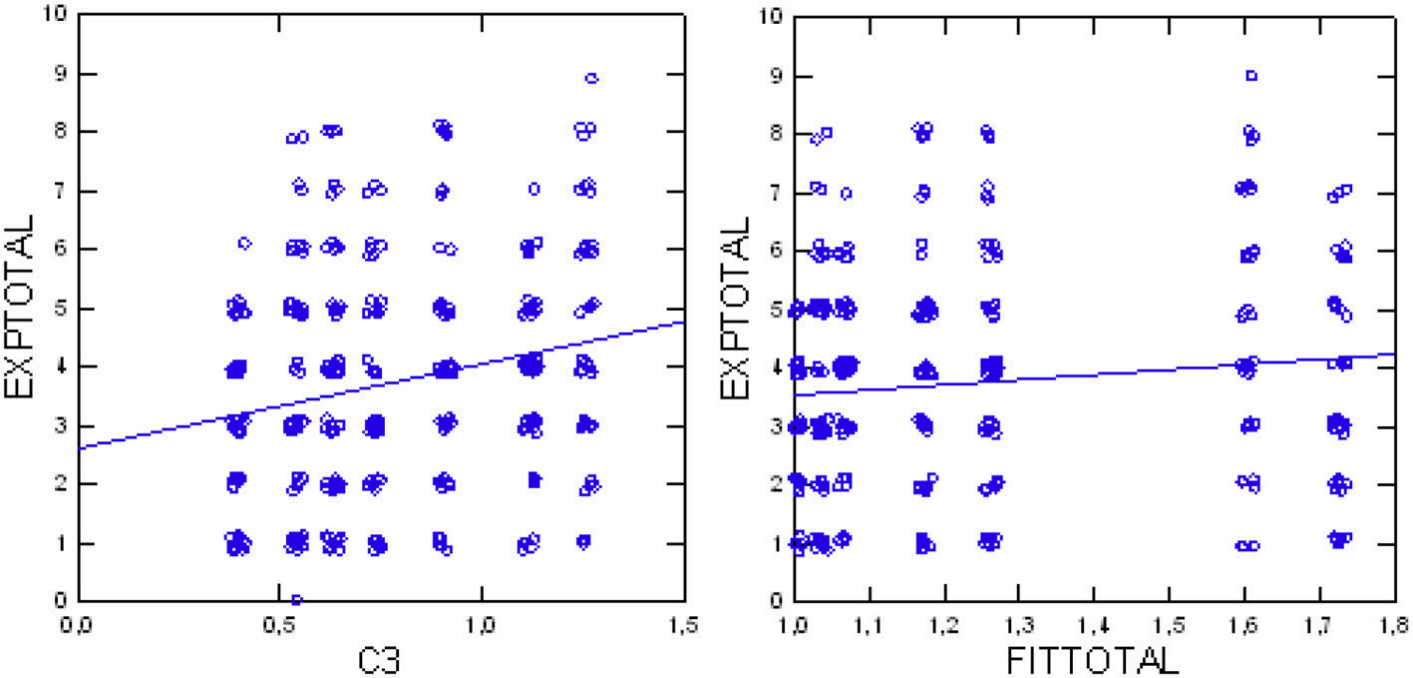
Total patients' experiences (EXPtotal) in relation to component C3 (continuity of care; **Left**); total patients' experiences (EXPtotal) in relation to degree of implementation (FIT64btotal) **(Right)**.

The statistical analysis (Jonckheere test, *df* = 1) verified our first primary working hypothesis: more patients' experiences were reported in departments with higher FIT64b grading, as shown by a significant increase of EXPtotal along with increasing FIT64btotal (*Z* = 2.82, *p* = 0.0048, deductive test; alpha = 2.5%). Follow-up analyses checked the influence of each single FIT64b component on the patients' experiences in an exploratory manner: Especially the components C3 (*Z* = 4.13, *p* < 0.0001), C5 (*Z* = 3.10, *p* = 0.0019), C8 (*Z* = 3.30, *p* = 0.0010) and C9 (*Z* = 2.63, *p* = 00.0085) yielded significant results (Table 3). Table 2 (upper part) presents test results for the FIT64b components of all seven departments, and Figure 2 illustrates the trend for the relationship between C3 and FIT64btotal.

**Table 3:** Relevance of FIT64b components for patients' total experiences + evaluations.

		**FIT64b total**	**C2**^a^****	**C3**^b^****	**C4**^c^****	**C5**^d^****	**C7**^e^****	**C8**^f^****	**C9**^g^****	**C10**^h^****	**C11**^i^****
FIT64btotal (**7 departments**)	m	**1.27**	2.83	**0.80**	2.64	**2.43**	0.55	**0.77**	**0.80**	0.76	1.00
	s	**0.29**	0.93	**0.31**	0.95	**1.10**	0.30	**0.15**	**0.25**	0.42	0.27
Trend EXPtotal (experiences)	Z	**2.82**	0.50	**4.13**	0.93	**3.10**	−1.89	**3.30**	**2.63**	−0.35	−0.48
	p	**0.0048**	0.61	**<0.0001**	0.35	**0.0019**	0.0590	**0.0010**	**0.0085**	0.72	0.63
Trend EVAtotal (evaluations)	Z	**2.85**	−0.17	**5.03**	0.19	**3.50**	−0.72	**2.90**	**2.67**	−0.36	−1.02
	p	**0.0440**	0.87	**<0.0001**	0.85	**0.0005**	0.47	**0.0037**	**0.0076**	0.72	0.31

*The table shows the sumscore and importance of each component. (7 departments; m, mean; std, standard deviation; Z and p by Jonckheere test DF = 1; significant findings indicated in bold font)*.

a *flexible care management across settings*,

b *continuity of care*,

c *multi-professional cooperation*,

d *therapeutic group sessions across settings*,

e *involvement of informal caregivers*,

f *accessibility of services*,

g *patient autonomy in steering of services*,

h *cooperation across sectors*,

i *growth of professional expertise*.

**Figure 3:**
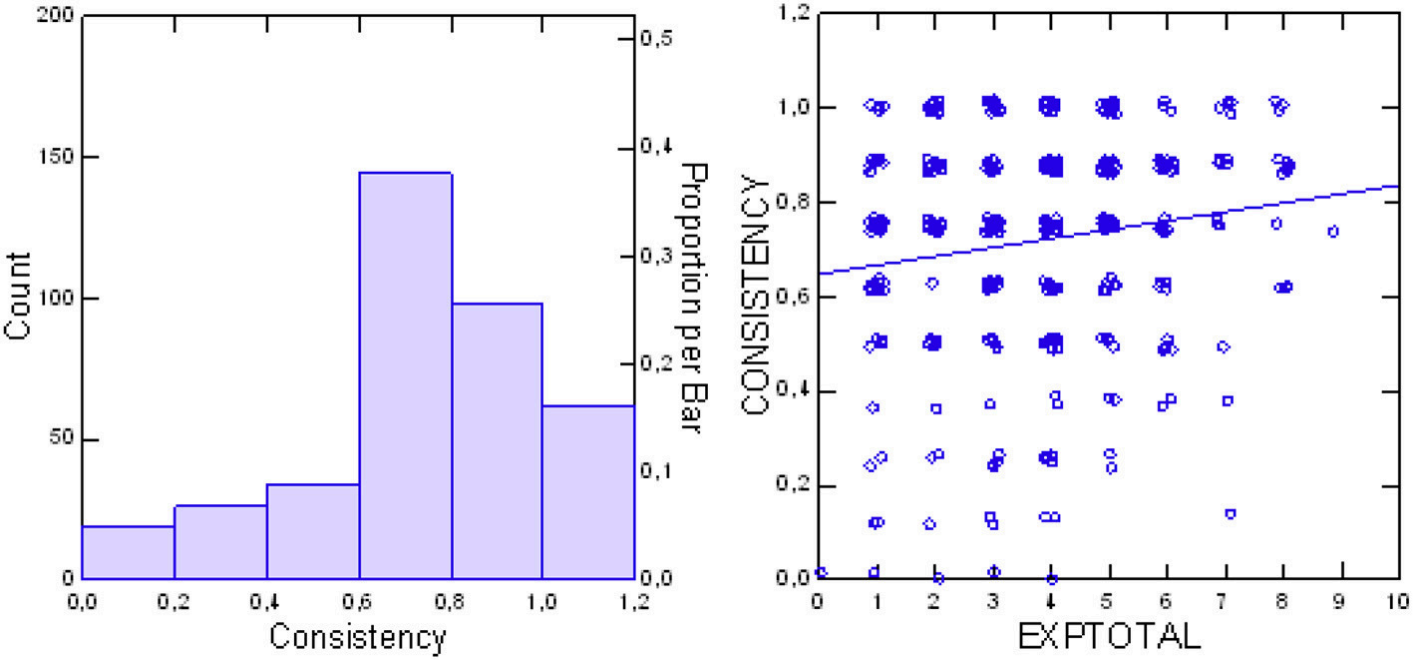
Distribution of SEPICC consistency scores **(Left)** and consistency of patients' assessments in relation to total patients' experience (EXPtotal) **(Right)**.

As mentioned above, the SEPICC uses contradictory questions to evaluate the consistency of patients' assessments. Within the possible range from 0 to 1, the group mean of 0.718 ± 0.244 indicates high consistency. The distribution of consistency scores (Figure 3) indicates that consistency of patients' assessments increased significantly with their increasing experiences with FIT64b components (Jonckheere exploratory test *df* = 1, *z* = 2.529, *p* = 0.0057).

##### Evaluations of patients

The second part of the SEPICC questionnaire is dedicated to patients' evaluations of FIT64b components. Descriptive statistics relating to the ten questions (Eva1-10) of this part of the questionnaire are presented in Table 4. In most cases, the mean scores were above 2, indicating positive assessment. Answers to questions 8 (Eva8) and 5 (Eva5) are the opposite to questions 3 and 10, which had the consequently expected lower scores. There was a negative evaluation for question Eva4, relating to the patients' perception of the quality of outreach care. Furthermore, the total evaluation score (EVAtotal) ranged from 0.211 to 4.00 with a mean of 2.65 ± 0.67, indicating a positive overall evaluation of the FIT64b models in the seven departments.

**Table 4:** Total patients' evaluations of FIT64b components (SEPICC part 2).

	**EVAtotal**	**Eva1**	**Eva2**	**Eva3**	**Eva4**	**Eva5**	**Eva6**	**Eva7**	**Eva8**	**Eva9**	**Eva10**
*N*	378	378	377	377	375	371	375	376	375	377	377
Mean	2.653	2.638	3.191	2.512	1.971	2.032	2.579	3.234	1.659	2.496	2.920
S	0.668	1.212	1.118	1.244	1.301	1.249	1.295	1.040	1.323	1.146	1.146

*The table shows the evaluative results of single components: The questionnaires questions Eva1, 2 & 7 refer to C3, the question Eva3 to C5, questions Eva4 & 9 to C6, questions Eva5 & 10 to C4, question Eva6 to C2, and question Eva8 to C5*.

**Figure 4:**
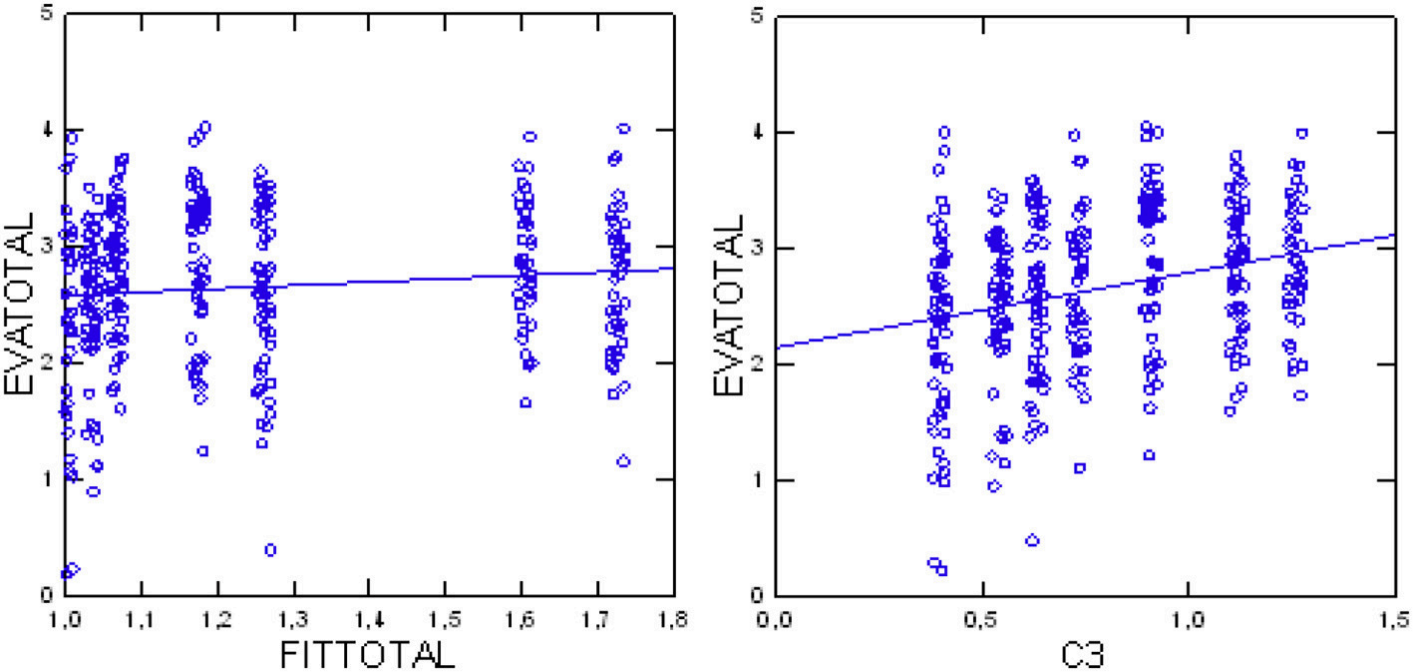
Total patients' evaluations (EVAtotal) in relation to component C3 (continuity of care; **Left**) and total patients' evaluations (EVAtotal) in relation to grade of implementation (FIT64btotal) **(Right)**.

The testing of the second primary null-hypothesis (Jonckheere test) indicated a significant positive trend (*p* = 0.0044, *df* = 1, *z* = 2.85) for higher evaluations (EVAtotal) with higher total FIT64b conformity in total (FIT64btotal), thus verifying the working hypothesis. Subsequent analyses tested the influence of each single FIT64b component on the patients' evaluations in an exploratory way: Again, the components C3 (*Z* = 5.03, *p* < 0.0001), C5 (*Z* = 3.50, *p* = 0.0005), C8 (*Z* = 2.90, *p* = 0.0037) and C9 (*Z* = 2.63, *p* = 0.0076) yielded significant results (Table 3). Table 2 (lower part) presents test results for each FIT64b component and Figure 4 illustrates the trend for a relationship between C3 and FIT64btotal.

**Figure 5:**
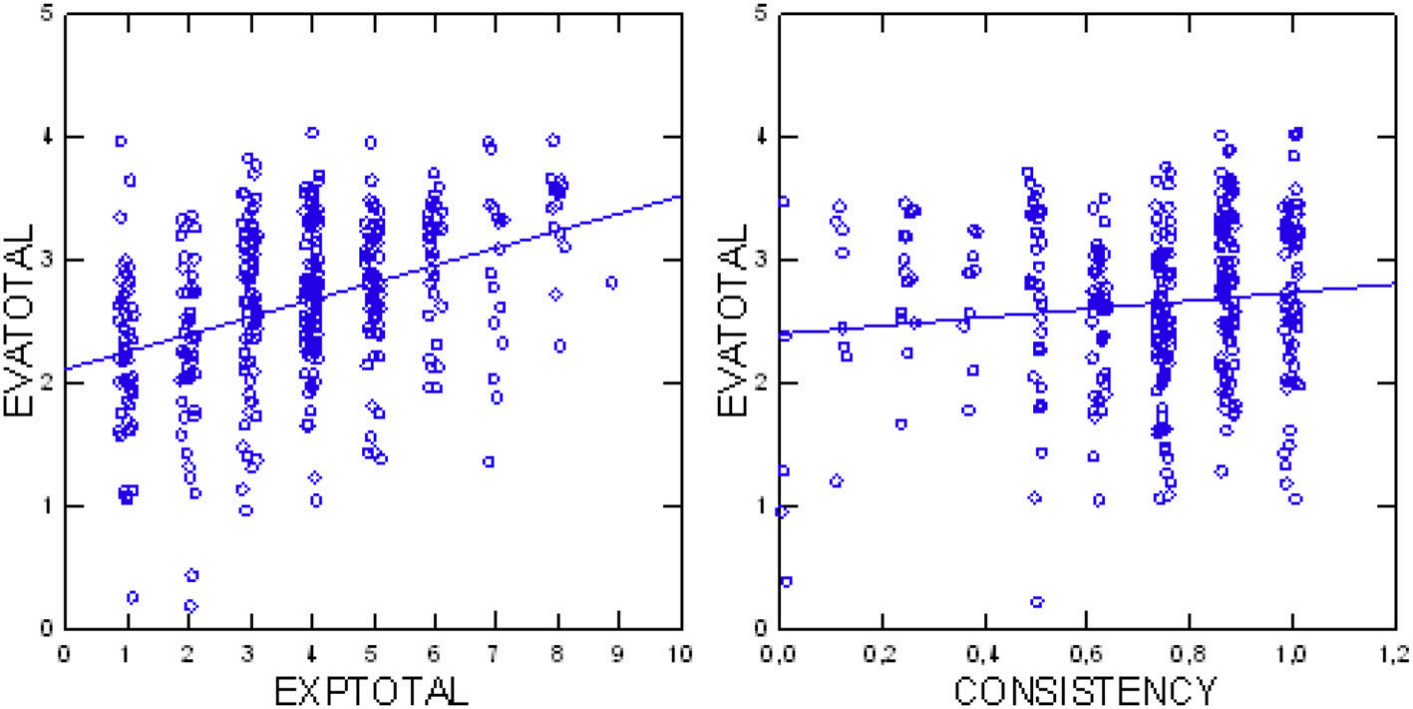
Total patients' evaluations (EVAtotal) in relation to their total experiences (EXPtotal) **(Left)**; total patients' evaluations (EVAtotal) in relation to consistency of assessments **(Right)**.

Analysis of the relationship between evaluations and experiences of patients indicated a positive correlation of increasing evaluations with increasing experiences (*p* < 0.0001 Jonckheere test, *df* = 1, *Z* = 7.621). Figure 5 illustrates this trend. Additionally, we found that evaluations were more positive with increasing consistency of the patient's assessments (*p* = 0.0259 Jonckheere test, *df* = 1, *Z* = 1.944; see also Figure 5).

Analyses of effects of long vs. brief durations of FIT64b activities in relation to patients' results was not productive, as the 64b project durations of all but one of the involved seven departments had implementation since more than 2 years.

#### Qualitative Analyses

Most patients were familiar with the structural and processual changes following the implementation of FIT64b models. The grade of implementation of FIT64b specific components was extensive, such that the components proved useful as deductive categories during qualitative analyses. High saturation of qualitative data was found for components C2, C3, C6, C8, and C9.

Continuity of care (C3) was highly valued according to qualitative assessments, being experienced as leading to “more trustful relationships” (FG_E1:10) between staff, patients and their kin as well as to a “more solid and nuanced understanding” (FG_V1:18, FG_V4:17) between them. Yet, continuity of care was also viewed critically by some patients due to its “potential to render the staff blind to the patients' developments” (FG_V1:17) as well in relation to personal data protection. Autonomous steering of services (C9) and flexible care management across settings (C2) were also associated in the qualitative data sets. They were both perceived to lead to more “need-adapted forms of treatments” (FG_V5:6; FG_V1:7f), especially according to the experiences of difficult to treat patients. Being able to “choose ones' own treatment setting” (FG_V5:6, FG_V1:7f) was perceived to increase “personal empowerment” (FG_V2:13, FG_V4:20, FG_V7:4).

Positive experiences of outpatient (C1) and residential (C6) forms of care corresponded to affirmative evaluations: Patients valued both these forms of care for their potential to deal with “embedded and real-life problems” (FG_V9:11, FG_V6:31, FG_V2:21), instead of receiving treatment “in a greenhouse” (= on a ward) (FG_Z5:7). Outpatient and residential forms of care were perceived to be “normalizing and de-stigmatizing” (FG_V1:16, FG_V7:9, I_Z2.1:5f, FG_V7:4), albeit at the risk of having the potential to “disrupt a person's or family's privacy” (FG_V2:15). Components C5 and C8 proved to be of little relevance according to qualitative analyses.

### Mixed Method Results

#### Methodological Impact of Components

As further discussed below, the specific FIT64b components were of high methodological value for the integration of the three study parts, i.e., quantitative assessments of patients, quantitative assessments of staff, and qualitative assessments of both patients and staff: First, the 11 components were the fundamental basis for all the study's main research questions and for the development of research instruments, that is to say the research guidelines for qualitative assessments, the instrument for quantitative patient assessment (SEPICC), and the core instrument for the quantitative staff assessment. Second, the FIT64b sum score allowed for grading and integration of results with different levels of implementation. Third, the components enabled interpretation of the experiences and evaluations of staff and patients and their interrelations equally for quantitative, qualitative and data sets collected as part of clinical routine.

#### Concordant and Discordant Results

The results of all three study parts demonstrate that higher scores for experiences and evaluations of both patients and staff experiences increased with the extent to which a FIT64b project had been realized, measured either according to the level of FIT64b-component implementation (assessments of patients) or according to the length of project duration (assessment of staff): Whereas the patients' experiences (EXPtotal) and evaluations (EVAtotal) increased significantly with the degree of FIT64b aggregate implementation (FIT64btotal), the staff evaluation scores were higher for departments with at least 2 years since implementation of FIT64b. Furthermore, we saw a plateau phase in the qualitative study part that was defined by more sophisticated FIT64b-related activities, such as attainment of expertise. On the other hand, changes were more likely to be perceived by both staff and patients during the early establishment phase, when a department's structures and processes were undergoing extensive alterations.

Further, the significance of most of the 11 FIT64b components was concordant between the different study parts: In both the qualitative and quantitative patient-related assessments, the components continuity of care (C3), accessibility of services (C8), and sovereign steering of services (C9) reached high saturation of data for the qualitative part, and significant correlations for the patients' experiences and evaluations in the quantitative part. In contrast, C5 (therapeutic group sessions across all settings) was of little significance to qualitative analyses, whereas the quantitative assessments of patients showed significant effects on experiences and evaluations. Similarly, C6 (outreach care) was a matter of contention in the qualitative assessments, while being of lesser importance for the standardized measurements.

## Discussion

### Discussion of Main Findings

This is the first multi-center study that provides for a mixed-method exploration of the implementation of FIT64b models for mental health care in Germany, which documents the roles of specific program components in experiences and evaluations of patients and staff. The main findings were robust in all three study parts, involving structural, qualitative, and quantitative data sets. Further, resent findings aligned with the results of the pre-study (29) in that all investigated departments experienced a relatively stable and narrow set of structural and processual changes upon implementing a budgetary capitation system according to §64b SGB V. Overall, rather rigid forms of mainly inpatient care shifted to more flexible and integrated types of outpatient and outreach treatment. During this process, there was a drastic reduction in hospital beds and wards or units either decreased in size or integrated with other units. New, more outpatient-oriented treatment structures or philosophies developed, which bore a relation to new attitudes, expertise, and practices among staff. Variants of these changes could be mapped between departments by the FIT64b program components that had been developed during the pre-study, thus serving to integrate the main inquiry's study parts (see section Methodological Discussion).

As shown in the main study's quantitative patient assessments, FIT64b-specific changes of structures and processes were more likely to be experienced in departments with a greater implementation. Thus, during quantitative assessments, the patients' perceptions depended on their department's level of development. A seemingly inverse relationship was seen during qualitative analyses, whereby structural or processual changes were more tangible for patients during the initial phase, and had declined in the plateau phase more than 2 years after introduction of the FIT64b project. However, this difference resulted from methodological disparities: Whereas quantitative assessments analyzed the *status quo* of the FIT64b implementation, the qualitative assessments evaluated the manner whereby the related structural and processual changes came about. Thus, both the qualitative and quantitative study parts concurred in demonstrating that the patients under investigation *perceived* FIT64b-specific changes, albeit differing in the way that kinds of perceptions were measured.

Further, not only the patients' experiences, but also their evaluations correlated with the degree of Fit64b implementation. The more the patients experienced (EXPtotal), and the higher the degree of implementation (FIT64btotal), the better were the patients' evaluations of the FIT64b program components (EVAtotal). Thus, higher levels of both implementation and patients experiences of FIT64b-specific changes predicted for higher degrees of approval. In other words, the more tangible the FIT64b related changes were for the patients, the more they were appreciated.

This main result of the quantitative assessments agrees with the qualitative analyses: during qualitative data collection, patients having extensive experiences with FIT64b-specific structural and processual changes generally approved of these new forms of treatment, whereas with fewer experiences remained more skeptical about changes. For instance, the value of outreach and home care (C6) was highly contentious, being a type of care with less readily appreciated benefits for those who had not experienced it directly. To give a second example, flexible types of care (C3) often initially caused confusion for both staff and patients, but, after full implementation, lead to greater satisfaction as they allow for more need-adapted forms of treatment. The general finding that concrete experience of new treatment structures or processes enhances their evaluations had been described elsewhere (39). Moreover, the association was robust in our study, as demonstrated by the large concordance of significances (quantitative assessments) with levels of saturation (qualitative assessments; see also sections Concordant and Discordant Results and Discussion of Components). The patients' evaluations proved to be reliable, with consistency scores during quantitative assessments increasing with increasing patient experiences and evaluations.

Contrasting with these patient-related results, the main results for the staff were less coherent: there was no significant correlation between the staff evaluations and the degree of implementation as measured by FIT64btotal. However, the staff evaluations were higher for departments with at least 2 years of history of FIT64b activities. This difference may at first sight seem surprising, but again methodological factors could be explanatory. The FIT64btotal represents the total score of all 11 components, whereas the project duration score relates to the overall duration of FIT64b activities at the department. Thus, the scores capture different aspects of the same phenomenon. Whereas the FIT64b score specifies the intensity of implementation, the project duration score relates to its temporal span. The latter seems to be more significant for the staff, representing the time it takes to get used to the structural and processual changes arising from FIT64b model implementation. This finding is consistent with a literature report showing that a longer career of the staff in CRT, ACT or CMHT services was associated with experiencing less emotional exhaustion and depersonalization in response to procedural changes (40). However, we note that nursing staff evaluated FIT64b-projects rather more negatively than did the medical staff and psychologists. This may reflect the burden of having to implement most of the structural changes central to the implementation of the FIT64b-projects. Furthermore, these changes seemed to be less transparent for nursing than for medical staff or psychologists, the latter usually being more involved in the theoretical aspects of new developments (41). The more negative assessment by nurses may reflect frustration due to their more passive role during the project development phase.

### Discussion of Components

Our study demonstrates that the effectiveness of current implementation of FIT64b programs in Germany is highly variable. Thus, and in contrast to other flexible and integrative forms of treatment such as ACT and CRT, there is no all-encompassing FIT64b model that may be generalized over sites. Consequently, the comparison of FIT64b models both among themselves and with international models is quite difficult. Instead, a productive strategy may be to compare the FIT64b critical ingredients with those of various international models.

In both qualitative and quantitative patient-related assessments, the FIT64b program components C3 (continuity of care), C8 (accessibility of services) and C9 (sovereign steering of services) attained high saturation of data in the qualitative part, while showing highly significant correlations for patients' experiences and evaluations in the quantitative part. In contrast, results concerning flexibility of treatment (C2), therapeutic group sessions across all settings (C5), and outreach care (C6) were inconclusive for both methodological approaches, either yielding high saturation during qualitative analyses, or being of high importance during standardized measurements.

According to a British survivor-controlled study (42), both continuity of care (C3; defined as good communication between staff and infrequent staff changes) and likewise accessibility of services (C9; defined as low waiting for services, being able to choose and to avoid services, and having assess to peer support) represent two fundamental facets of supportive forms of care. Staffing continuity is a critical program ingredient for ACT because ACT uses a team approach for serving clients with severe mental illness in community settings (43, 44). On the other hand, failure to achieve full and continuous staffing can result in interrupted services, reduced quality, and diminished support for clients (45). Similarly, a recent study of stakeholders' views on critical components and implementation of CRT and HT in England suggested that continuity of care should be prioritized in service improvements (46). Indeed, continuity of care is widely considered to be a central indicator of successful, integrated community services (47–49). Ongoing care increased the likelihood that patients would recommend their clinic to others (49). Finally, a number of official inquiries into suicides and homicides by psychiatric patients suggested that a lack of continuity of care may have been a central factor in these catastrophic outcomes (50, 51).

In international guides of mental health policy implementation, CRTs were traditionally described as gatekeepers to mental health services, providing rapid assessment of peoples' needs and (where appropriate), immediate multi-disciplinary home treatment 24 h a day, 7 days a week (52). The importance of this critical treatment ingredient is emphasized by the fact that CRTs, having created effective access to mental health care for adults, continuously expand this access at the expense of other age groups, i.e., the elderly and children (53). During a concept-mapping procedure across five European countries, accessibility of services (C8) was found to be a highly important component of good community care for people suffering from severe mental illness (54). The importance of coordinated services, which are easily accessible for the care network, including case managers and family physicians, was also highlighted in a Canadian study (55). Further, the geographical accessibility of services independently contributes to reducing the duration of untreated psychosis and is one of the major factors leading to treatment delays in more remote areas (56).

In contrast to the results of quantitative assessments, the category C2 (flexibility of services) was highly saturated for the qualitative analyses of our study, even exceeding the saturation of C9 (autonomous steering of services), which proved to significantly influence the patients' evaluations of FIT64b components. Yet, from a qualitative perspective, C2 is thematically closely linked with C9, demonstrated by the fact that the related codes and sub-categories of the qualitative analyses of both sections were almost interchangeable. Thus, in the patients' evaluations, the flexibility of FIT64b-related structures and processes (C2), was directly linked with aspects of free choice of treatment options and their sovereign adoption (C9). This interrelation is affiliated with international research on critical ingredients of FIT programs, where new forms of flexible and individualized care are closely related to the elements of patient choice and autonomy (46). Thus, the lack of significance of C2 in the quantitative assessments of our study might reflect difficulties in its operationalization.

The significance of C5 (therapeutic group sessions across settings) likewise differed between the qualitative and quantitative study parts, again plausibly due to differences in the underlying methodological approaches. During qualitative analyses, codes and categories under the section C5 were sparsely reported. Instead, responses tended to assemble under section C3, which is the main code for “continuity of care.” Thus, components C5 and C3 seem to be strongly interlinked, with both describing continuous forms of care, one more in relation to groups and the other more related to individual patient-staff relationships.

Finally, qualitative and quantitative results of our study showed high disagreement concerning the evaluation of outreach care (C6). Various forms of international FIT models consider home and outreach treatments to be an integral part of their practices (46, 56, 57). In our sample, outreach care had a relatively low statistical importance compared to other program components. This reflects the traditionally scant development of outreach care in Germany. Until the passage of a special law in 2016 (58), home treatment could only be adequately implemented within German mental health care on the legal basis of §64b SGBV. On the other hand, all experts and most of the participants within qualitative investigations have agreed that this component should be strongly developed in the future through FIT64b models and that its availability represents a good indicator for the quality of these forms of care.

### Methodological Discussion

Our study demonstrates the high value of a mixed methods approach, wherein several results were robust over all three study parts. The use of various methodological approaches thus served to (in a manner of speaking) triangulate our results. While there were some disparate findings between the three study parts, we feel that these differences should not be viewed as mere inconsistencies, but a representation of different perspectives on the same phenomena. Care situations are inherently complex (24), and different analysis methods probe distinct aspects of the whole. The partially divergent results in mixed method evaluations do not thus invalidate the approach, but rather lend greater credence.

The developed list of program components proved to be useful in many ways. First, this approach allowed for an integrated process of data collection by laying a common foundation for most of the employed research instruments. Second, the components facilitated the integration of data analyses by developing into main categories during the qualitative analyses and guiding the major research questions for the quantitative assessments of both staff and patients. Third, the components allowed for an integrated process of interpreting and representing data, thereby enabling a parcellation of results of the various parts of the study. In brief, concordances between data sets served to cross-validate results, whereas discordances revealed issues needing further examination.

### Limitations

As discussed above, results partly differed between the study parts. Since these differences arguably represent various aspects of the gestalt, they were heuristically useful in understanding the multi-faceted practices of FIT64b projects. Further, although the sample of FIT64b projects involved was relatively large, it may not be sufficiently representative. Our original aspiration for a sample of 600 patients proved to be unattainable given our limitation for 1 year of data collection. We did implement other approaches to sample a representative patient-strata, for instance by basing our sampling process on a randomized design. Further, we employed in this study a cross-sectional design, which enables the assessment of past exposures to FIT64b models, but has limited capacity to determine causality. Another potential limitation was our use of self-reporting for some of measures such as experiences, assessment, and satisfaction, which is generally associated with a risk of bias (59). Finally, there was a substantial drop-out rate, both of departments and of data (see Figure 1).

### Practical Implications and Direction for the Future

Our findings are hypothesis-generating and inductive in nature, thus requiring further testing and development aiming to improve clinical practice. Based on present and other preliminary results, we are currently developing a larger multi-center cohort study (“PsychCare”). This subsequent study will combine both qualitative and quantitative methods in a prospective and controlled design to generate both outcome- and component-related data of German FIT64b care models.

Our study demonstrates that structural and procedural changes in accordance with §64b SGB V are well-perceived and evaluated by patients and most staff. Indices of approval increased with levels of implementation and with greater duration, substantiating FIT64b treatment models as a legitimate alternative to standard forms of psychiatric care. FIT64b care models have been introduced by the German government with the explicit intention to generate evidence for motivating further reforms of the national psychiatric care system (21). In this context, results of our study can inform policymakers about further directions for elaboration of the reform process.

Besides this crucial role within the refinement of the German health care system, our study may contribute to further improve both national and international FIT-models. In this context, current and future FIT64b models should be scrupulous about implementing and evaluating continuous forms of care and accessibility of services, as these program components both had pronounced influence on the quality of the treatment models. Further, flexible and individualized types of care seem to positively correlate with patients' wishes for autonomy and choice of treatment, suggesting that particularly those components of FIT64b-models may require special emphasis.

Our data suggest that it is of the utmost importance to integrate all groups of staff into the processes of change. Traditionally, it is the academic staff who conceive of and implement new forms of treatment, whereas it falls to subordinate staff to “carry out” these ideas, thus playing a more passive role within the process. This may account for the lower satisfaction reported by nursing staff, who spend most of their time with the patients, and should thus properly be more actively integrated within the steps of system planning and legislation. Finally, this study draws attention to the need for expanded home and outreach forms of care in Germany, as these components emerged from our qualitative analyses as a good indicator for the quality of FIT64b models.

## Availability of data and material

The datasets underlying the current study are not publicly available due to the used data protection declaration and the nature of qualitative interviews where individual participants could be possibly identified. Parts of the data set are available from the research group on reasonable request.

## Author Contributions

SvP and YI wrote the first draft of the manuscript. MH, JT, and JJ modified successive drafts. JT was mainly responsible for the statistical analysis. SvP, MH, and YI contributed to study design. All authors contributed to and have approved the final manuscript.

### Conflict of Interest Statement

The authors declare that the research was conducted in the absence of any commercial or financial relationships that could be construed as a potential conflict of interest.

## Abbreviations

CMHT: Community Mental Health Treatment
CRT: Crisis Resolution Teams
ACT: Assertive Community Treatment
ICM: Intensive Case-Management
HT: Home-Treatment
FIT: Flexible and Integrative Treatment Models
SGB-V: German Social Code V
FAÄ: Questionnaire on Working Situation for Doctors
RN4CAST: Registered Nurses Forecast
SEPICC: Scale for Evaluation of Psychiatric Integrative and Continuous Care
SCL-90-R: Symptom Checklist 90
EXPtotal: Trends of Experience with FIT (Summarized Patient's Experience score)
EVAtotal: Trends of Evaluation of FIT Experiences (Summarized Patient's Evaluations of FIT Experiences)
FIT64btotal: Degree of Implementation of FIT.
